# Assembly and Function of Seed Endophytes in Response to Environmental Stress

**DOI:** 10.4014/jmb.2303.03004

**Published:** 2023-05-30

**Authors:** Yong-Lan Wang, Han-Bo Zhang

**Affiliations:** State Key Laboratory for Conservation and Utilization of Bio-Resources in Yunnan, Yunnan University, Kunming 650091, P.R. China

**Keywords:** Seed endophytes, biotic stresses, abiotic stresses, assembly, growth-promoting mechanism

## Abstract

Seeds are colonized by diverse microorganisms that can improve the growth and stress resistance of host plants. Although understanding the mechanisms of plant endophyte-host plant interactions is increasing, much of this knowledge does not come from seed endophytes, particularly under environmental stress that the plant host grows to face, including biotic (*e.g.*, pathogens, herbivores and insects) and abiotic factors (*e.g.*, drought, heavy metals and salt). In this article, we first provided a framework for the assembly and function of seed endophytes and discussed the sources and assembly process of seed endophytes. Following that, we reviewed the impact of environmental factors on the assembly of seed endophytes. Lastly, we explored recent advances in the growth promotion and stress resistance enhancement of plants, functioning by seed endophytes under various biotic and abiotic stressors.

## Introduction

We know that there are a large number of endophytes in the tissues of plant roots, stems, leaves, flowers, fruits, and seeds [1-4]. As a reproductive organ of plants, seeds are one of the most significant stages in the life history of plants [5] and carry a variety of endophytic microbes that can be transmitted to offspring through parental plants [6]. Endophytes present in seeds do not produce any visible disease symptoms on seeds or host plants and are classified as both obligate and facultative [7]. Here, this classification depends on their life strategies: the survival of obligate endophytes depends entirely on the host plant, while facultative endophytes can survive outside the host plant and are not completely dependent on the host plant [8]. As a key component of the plant microbiome, the seed microbiome is not only the end point of microbial community assembly in seeds, but also the starting point of new seedling microbiome assembly [9]. Generally, seeds and endophytes form a symbiotic relationship by which endophytes colonizing the seed interior depend on the host for the required water, nutrients, and oxygen [10]; in turn, endophytes can enhance seed viability, improve germination and resilience, and directly or indirectly promote plant growth and development. Under stress conditions, endophytes can also improve the growth of host plants. Therefore, seed endophytes are of ecological importance for host plants [11, 12].

Seed endophytes are phylogenetically diverse, and the common phyla are fungi of Ascomycota, and Basidiomycota, and bacteria of Firmicutes, Proteobacteria, Bacteroidetes and Actinobacteria [13, 14]. For seed endophyte bacteria, the common genera in different host plant seeds are *Bacillus*, *Paenibacillus*, *Pantoea*, *Pseudomonas*, *Staphylococcus*, *Micrococcus*, and *Acinetobacter* [11]. Seed endophytic fungi are dominated by Basidiomycetes and Ascomycetes [15]. Ascomycetes are more diverse, such as Dothideomycetes, Sordariomycetes, Leotiomycetes, and Eurotiomycetes, and the Basidiomycete class Tremellomycetes dominates the seeds of Brassicaceae plants [16]. Dothideomycetes are a kind of filamentous ascomycete, such as *Alternaria*, *Cladosporium*, *Phoma*, *Phaeosphaeria*, and *Epicoccum*, which are common genera [15]. Of course, *Epichloae* species are the most common seed endophytic fungi in the Poaceae family [17]. Even so, studies have noted that the diversity of endophytes in plant seeds is generally lower than that in other tissues [18]. In fact, the long-term evolutionary selection of plants and the repeated transmission of endophytes from generation to generation, together with the limitations of the internal environment of seeds, usually lead to a decrease in endophyte diversity and even the loss of beneficial endophytes [19]. Although endophytes in seeds have been continuously discovered by means of high-throughput sequencing techniques, some endophytes are difficult to isolate and culture from seeds even if they show high abundance [1, 20, 21]. These limitations bring challenges to research on seed endophytes, and thus, the effects of most seed endophytes on plants have yet to be clarified [22].

Recently, seed endophyte-host interactions in response to environmental factors have gained increasing attention. Plant-seed microbe interactions in response to climate change are extremely important [23]. In particular, the increasing frequency of droughts and extreme events has severely affected crop yields and constrained agricultural development [24, 25]. Therefore, the potential of using seed endophytic microbes to improve crop yields in the context of adhering to sustainable agricultural development has repeatedly attracted attention [26]. Moreover, with the development of industrialization, soil environmental pollution has become an urgent problem, especially the soil acid-base imbalance caused by heavy metal pollution. Phytoremediation can effectively degrade toxic contaminants in soil through interroot microbial degradation [27]. Interestingly, in contaminated soils, a recent study indicated that some species or populations of seed endophytes can also show eco-physiological responses to withstand stress factors by affecting the activities of antioxidant enzymes in host plants [28].

Many excellent papers have reviewed the role of endophytic microbes associated with roots, stems, leaves, and flowers in plant growth and adaptability [29-31], and endophytic microbes have been commonly applied as inoculants for crops [32, 33]. Nonetheless, the understanding of the assembly and function of seed-associated endophytes in response to environmental stress remains to be reviewed. Here, we first provided a framework for the assembly and function of seed endophytes and discussed the sources and assembly process of seed endophytes; then, we reviewed the impact of environmental factors on the assembly of seed endophytes; and finally, we explored recent advances in the growth promotion and stress resistance enhancement of plants, functioning by seed endophytes under various biotic and abiotic stressors.

## Framework for the Assembly and Function of Seed Endophytes

Plant endophytic microbes have two transmission modes: horizontal transmission and vertical transmission. Although their relative importance is still unclear, both modes of transmission may contribute to the growth of host plants [34]. Horizontal transmission refers to endophytic microbes obtained through airborne spores (air source), through foraging and transferring of insects, birds, and other animals (animal source), and through rhizosphere soil (soil source); vertical transmission means that endophytic microbes are the source of the transfer of seeds, pollen, etc. These vertically transmitted endophytic microbes are mainly obligate symbionts because their survival is completely dependent on the host plant [6, 35]. In fact, we know that the vertical transmission of endophytes is more common than previously expected and that beneficial microbes colonizing in seeds are usually transmitted from generation to generation. Therefore, vertical transmission is also defined as a direct transfer from parents to offspring because it is conducive to reciprocal symbiosis [36]. For example, in the study of Hodgson *et al*. [6], two endophytic fungi, *Alternaria alternata* and *Cladosporium sphaerospermum* were vertically transmitted from mother to offspring in six species of Gramineae: *Centaurea cyanus*, *Centaurea nigra*, *Papaver rhoeas*, *Plantago lanceolata*, *Rumex acetosa*, and *Senecio vulgaris*. In addition, the same endophytic microbial species were detected in *Panicum virgatum* L. seeds and in plants of seed origin collected one year ago, confirming that endophytes are transmitted vertically to the next generation of hosts [37]. Several studies have even noted that the vertical transmission rate of endophytes is more than 90% [38], most of which represents the core microbiota within the seeds.

Generally, endophytes have different pathways to enter seeds, and in the studies that have been described, there are three routes of microbial transmission into seeds: (1) through the use of nonvascular tissues or xylem; (2) through the stigma of the parent plant; and (3) through contact between the seed and external sources of contamination [11, 16, 39, 40]. The ‘primary symbiont hypothesis’ suggests that seed endophytic microbial transmission is the result of interactions between plant defenses and seed-borne microbes, which cause a primary or at least a unique endophytic symbiont in an individual plant seed [41]. Therefore, the stage of seed germination to growth is influenced by the presence or absence of this primary symbiont, such as helping plants adapt to competition; consequently, the core microbiota in plants may be endophytic microbes transmitted by the mother [41, 42].

**Fig. 1:**
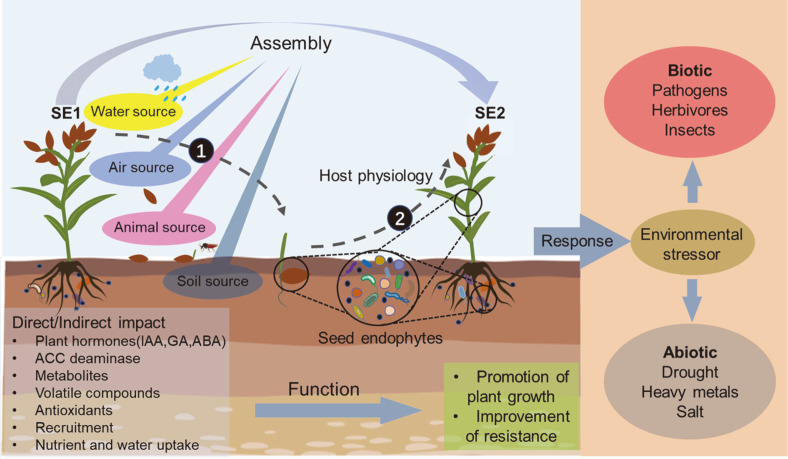
Framework for the assembly and function of seed endophytes in response to environmental stress. Seeds naturally fall off to the ground after maturation (arrow 1) and then germinate to grow until flowering and fruiting to produce the next generation’s seeds (arrow 2), in which seed microbes from generation one (SE1) can partially pass to the next generation (vertical transmission), with other microbial sources including soils, air and animals (horizontal transmission) to build the seed microbes in generation two (SE2). The assembly of seed endophytes is affected by host physiology during individual development, as well as by environmental stress including biotic (*e.g.*, pathogens, herbivores and insects) and abiotic (*e.g.*, drought, heavy metals and salt) factors and in turn, seed endophytes colonizing inside the host plant can promote plant growth and enhance stress tolerance, either directly or indirectly.

Therefore, we can conclude the framework for both the assembly and function of seed endophytes in response to environmental stress (Fig. 1). Seeds naturally fall off to the ground after maturation (arrow 1) and then germinate to grow until flowering and fruiting to produce the next generation’s seeds (arrow 2), in which seed microbes from the first generation (SE1) can partially pass to the next generation due to host physiological selection (vertical transmission), with other microbial sources including soils, air and animals (horizontal transmission) to build the seed microbes in the second generation (SE2). Undoubtedly, the assembly of seed microbiota from generation to generation can be affected by environmental stress that the plant host grows to face, and in turn, endophyte microbes colonizing inside the seeds can promote plant growth and enhance stress tolerance, either directly or indirectly. Here, the environmental factors are diverse and include biotic factors (*e.g.*, pathogens, herbivores and insects, etc.) and abiotic factors (*e.g.*, drought, salt, heavy metals, rainfall, agrochemicals, soil, heat, etc.). Currently, few studies address the response of seed endophytes to these environmental stressors.

Here, we emphasized the role of host physiology and environmental factors in the assembly of seed endophytic microbiota during their transmission from SE1 to SE2, which may lead to a change in the diversity of seed microbiota. Because host genetics play an important role in seed phenotype and chemical composition within seeds, parental lines may indirectly impact the plant-to-seed transmission stage of seed microbes and thus can influence seed endophyte assembly [43]. Therefore, there are four main processes that drive microbial community assembly and composition in host plants: (1) abiotic and host filtering; (2) ecological drift; (3) dispersal; and (4) species interactions [44-46]. A study by Kim *et al*. [47] confirmed that the distribution of rare seed microbe taxa is limited by dispersal, while the distribution of prevalent taxa is influenced by homogeneous selection and ecological drift. It has been proposed that the factors driving the assembly of seed microbiota may be based on niche ecological processes or neutral processes, usually stochastic in nature, with ecological niche processes involving the internal environment of the seed, the soil environment in which it grows, and recruitment, while neutral processes include obtaining microbes that are transmitted to the seed through air and rainfall [9]. Indeed, the neutral model also emphasizes the importance of selection as the main driver of microbial community assembly in different plant species, as well as the main ecological process for the succession of dominant taxa during seed filling and maturation [48]. In addition, it was found that the temporal colonization patterns of seed microbial communities were affected by niche changes and neutrality in the process of dynamic transmission from parent to progeny [49].

Prado *et al*. [50] showed that insect pollination is involved in the assembly of seed microbiota, a neutral ecological process of microbial transmission between flowers and seeds, and that it can reduce the diversity of seed-associated microbiota. In a recent study, Chesneau *et al*. [51] found that, during the development of individual seeds, the assembly of bacterial communities associated with bean seeds was mainly driven by selection, whereas ecological drift occurred in the early assembly stages of the radish seed microbiota, and the main process of community assembly shifted to be driven by selection in late seed maturation. In general, the enrichment of bacteria was reduced, and only one dominant taxonomic unit was able to colonize. Although some studies have observed *Raphanus sativus* in three successive generations of radish seeds, the low heritability of the seed microbiota over generations may be explained by climatic conditions leading to differences in community structure in successive generations of plants based on neutral processes. However, for the formation of seed-associated bacterial communities, ecological drift is an important driver, while propagation is also involved in the seed microbiota assembly of the fungal fraction of the seed microbiome [52]. Several studies have also shown that the number and diversity of culturable endophytes in seeds decrease with seed age [53, 54]. Furthermore, Abdelfattah *et al*. [43] referred to dormancy as the period between seed maturation and germination, including seed storage, seed banks, and the period the seed remains in the environment or inside the animal’s gut. In this state, multiple environmental stressors may also affect the assembly of second-generation seed endophytes (Fig. 1).

## Assembly of Seed Endophytes in Response to Environmental Stress

Many studies have explored the function of seed endophytes in plant growth and adaptability (see below discussion), and relatively few reports have determined the impact of environmental factors on the assembly of seed endophytes.

### Heavy Metal Contamination

Truyens *et al*. [55] reported the effects of contaminants on the composition and characteristics of seed bacterial communities associated with *Arabidopsis thaliana*, and found that cross-generational Cd exposure under greenhouse conditions resulted in changes in *A. thaliana* seed endophytic populations: *Sinorhizobium* sp. and *Micrococcus* sp. were dominantly present in the uncontaminated seeds, while *Pseudomonas* sp., Bosea sp., and *Paenibacillus* sp. were present in the Cd-exposed seeds. However, in another study, endophytic isolates from *Agrostis capillaris* seeds grown on uncontaminated soil (*i.e.*, control) were more numerous than those from contaminated soil, without much variation in species composition [56]. Moreover, Croes *et al*. [57] reported that the root endophytic communities of *Brassica napus* L. grown in Cd, Zn, and Pb-contaminated fields and noncontaminated fields showed a similar pattern for the common obligate endophytic community of seeds. Therefore, although heavy metal contamination may have an impact on seed-associated microbiota, host plants likely harbor similar core seed microbes, suggesting strong host selection pressure.

### Stalt Stress

Salt stress can greatly affect the assembly of seed microbiota of the host genotype and physiological adaptations of rice. For example, recent evidence suggests that the overall seed endophytic communities of salt-sensitive and salt-tolerant rice varieties are *Pantoea*, *Pseudomonas*, *Microbacterium*, *Kosakonia*, *Xanthomonas*, *Stenotrophomonas*, *Delftia*, *Ralstonia*, *Sphingomonas*, *Rhizobium*, *Paenibacillus*, *Herbaspirillum*, *Enterobacter*, *Curtobacterium*, and *Bacillus*. Under salt stress, the dominant community for both salt-sensitive and salt-tolerant rice shifted to a bacterial community dominated by *Flavobacterium*, *Pantoea*, *Enterobacter*, *Microbacterium*, *Kosakonia* and *Curtobacterium* [58].

### Drought Stress

The seed microbial composition can change with the exposure of parent plants to drought. A recent study showed that the structure of the common bean (*Phaseolus vulgaris* L.) seed microbiome (bacteria, archaea, and fungi) greatly varied under abiotic treatments (control; 66% moisture: mild drought; Hoagland nutrient treatment). Nutrition and intensive drought treatments changed the microbiota structure of bacteria and archaea communities; for the fungal community, *Aspergillus* fungal communities were dominant in both control and treated seeds, whereas other fungal taxa, *e.g.*, *Penicillium* and *Wallemia*, shifted in all treated seeds [59]. Furthermore, the bacterial endophytes of wheat seeds changed under drought conditions, with increased α and β diversity, and enrichment of Actinomycetes but significant depletion of Gammaproteobacteria. Nonetheless, when wheat is exposed to rainfed or drought conditions, drought-tolerant varieties selectively enrich some beneficial seed microbes [60].

### Cross-Generation Effects

Interestingly, Kim *et al*. [49] showed that parental seeds and the inner layer of the stem at the mature stage are the main microbial sources of the next generation’s seed. Therefore, it can be expected that, the environmental conditions for the storage of parental seeds and the whole process from parental seeds to the next generation of seeds can impact the origin of seed microbial communities (Fig. 1). For storage of parent seeds, there is evidence that temperature change, seed water content, and time interaction all have negative effects on the viability of endophytes [61]. A recent study showed that soil water stress in the parent environment will lead to a decline in the seed germination rate and overall seedling performance [62]. However, it is unclear how this stress impacts the origin of endophytes in the next generation’s seeds. In addition, pathogens and metabolic disorders in seeds also affect the assembly of seed microbes, and further impact their transmission to next generations [43].

Several points are worth exploring in the future for the assembly of seed endophytes in response to environmental stress. First, the host plant commonly faces diverse environmental stressors during the whole growth season (Fig. 1). Current works have explored the assembly of seed endophytes under single factors, such as heavy metal contamination, salt stress, and drought conditions, and a great challenge is to determine the compound environmental effects on the origin of seed microbial communities. Moreover, there is currently no literature to determine how seed endophytes respond to some popular novel pollutes, *e.g.*, microplastics. Second, by our review, we found that most research works have mainly focused on crops of host plants, particularly those from Poaceae (Table 1). Crops are under intense artificial management, which can generate strong selection pressure on seed endophytes, including using herbicides [63] and genetic modification of host plants [64]. However, there is no report exploring how artificial management impacts the seed endophyte composition interacting with environmental stress. Such works are very valuable for better application of seed microbes in sustainable agriculture. Third, little work has been performed to explore the response of seed endophytes to environmental change for those living in the wild. Seed endophytes can impact seed quality and viability thus influencing plant population dynamics [12]. For example, seed endophytes can prevent the loss of biodiversity and save threatened host species in ecosystem restoration [65]; thus, seed endophyte assembly is becoming important for understanding ecosystem stability under environmental change. Finally, it is not enough to explore intergenerational transmission [52] under environmental stress to better understand the coevolution of endophytes and host plants.

## Functions of Seed Endophytes in Response to Environmental Stress

The interaction between seed endophytes and plants is complex and diverse. In recent years, we have a more comprehensive understanding of the growth-promoting and stress-resistant mechanisms of seed endophytes under various biotic and abiotic environmental stresses, including: (1) promoting host plants to obtain nutrients by producing hormones; (2) producing metabolites and volatile compounds and harnessing lipopeptide genes to induce host defense against biological diseases; and (3) enhancing antioxidant activity (Table 1).

### Promotion of Plant Growth by Seed Endophytes

**Producing plant hormones to regulate plant growth.** Seed endophytes can directly promote plant growth by producing plant hormones or hormone-related signaling molecules (Fig. 1). Previously, many studies indicated that, endophytic bacteria in other plant tissues, such as *Azospirillum lipoferum*, can produce gibberellin and combine with other plant hormones to alleviate the drought effect of maize [66]; moreover, endophytic fungi such as *Phoma glomerata* LWL2 and *Penicillium* sp. LWL3 were also able to produce gibberellins, but they were isolated from plant roots, not seeds [67]. Although the mechanism of action to positively affect the host plant relies on multiple aspects, there is growing evidence that seeds require the involvement of phytohormones at all stages from germination to growth and maturity; therefore, seed endophytic-microbes can synthesize phytohormones in a direct or indirect manner to promote host plant growth [68]. Endophytic bacteria such as *Bacillus amyloliquefaciens* isolated from rice seeds could produce gibberellin; inoculation of *B. amyloliquefaciens* at the seedling stage significantly promoted rice plant height, mainly because endophytic bacteria could regulate the endogenous plant hormone gibberellin [69]. Of course, some seed endophyte-mediated growth-promoting mechanisms of plant phenotypes have not yet been explained. For example, inoculation with appropriate concentrations of seed endophytic fungi such as *Cladosporium cladosporioides* could significantly increase the germination rate of the coastal plant *Suaeda salsa*. Furthermore, it was found that *C. cladosporioides* also had an obvious growth-promoting effect on the growth of American sweetgum seedlings. However, the mechanism of promoting growth is unclear, and it is assumed that the fungus may secrete some kind of molecules involved in the signal transduction of plant hormones [70].

Seed endophytes have a strong ability to produce auxins such as indoleacetic acid (IAA). For example, 33% of the endophytes cultured from Cucurbitaceae seeds can produce the plant hormone indole-3-acetic acid; moreover, these endophytes can improve host nutrient acquisition by a variety of pathways, including nitrogen fixation, phosphorus solubilization, secretion of iron carriers, and production of ACC deaminase. Therefore, these strains can be developed into growth-promoting bacteria [71]. In addition, *Bacillus subtilis* HYT-12-1 in tomato seeds exhibited a significant plant growth promoting (PGP) effect on tomato seedlings by producing ACC deaminase to inhibit ethylene production, as well as enhancing nitrogen fixation and organic acid production [72].

**Improving the ability of plants to absorb nutrient elements.** Seed endophytes can improve the absorption capacity of nutrient elements to promote plant growth (Fig. 1). Seed endophytes can help plants obtain nutrients by dissolving phosphate [71], producing organic acids [72], fixing nitrogen [73], and producing iron carriers [74]. A recent study showed that the seed-borne beneficial microbe *Burkholderia gladioli* can promote nutrient uptake from the soil by the host plant when the plant is deficient in nutrients and can therefore be seen as a functional compensatory pool for plant rhizosphere colonization [75]. In addition, there are genes related to nitrogen and carbon fixation, photosynthesis, and oxidative phosphorylation in endophytes dominated by *Methylobacterium* in *Crotalaria pumila* seeds, and the genes involved in nitrogen metabolism can help host plants absorb nitrogen [76, 77]. Endophytic microbes in cactus seeds can release a large number of nutrients useful to plants from rocks (such as phosphorus in crushed rocks), produce volatile and nonvolatile organic acids, and have the ability to fix nitrogen [73]. In addition, some seed endophytes are able to secrete small-molecule proteins that bind Fe in the soil to promote Fe uptake by plants. For example, most strains of *Kosakonia* sp., *Pantoea* spp., and *Xanthomonas* sp. in rice seeds can produce Fe carriers and solubilize phosphate [74].

### Improvement of the Resistance of Plants to Biotic Stress

**Resistance to pathogens.** Several studies have shown that seed endophytes can improve plant resistance to diseases (Fig. 1). For example, the rice seed endophytic bacterium *Sphingomonas melonis* can secrete the small extracellular signal molecule o-aminobenzoic acid in coordination with the host response to prevent infection and the production of toxins by the pathogen *Burkholderia plantarii*, further clarifying the role of seed endophytes in plant pathology in the “disease triangle” [78]. Members of the genus *Pseudomonas* spp. isolated from the seeds of *Phragmites australias* secrete unknown antifungal metabolites to inhibit the fungal pathogens *Sclerotinia homoeocarpa* and *Fusarium oxysporum*. Interestingly, *Pseudomonas* spp. were previously shown to produce metabolites such as hydrogen cyanide and pyoluteorin for biological disease control; at the same time, *Pseudomonas fluorescens* (*i.e.*, Sandy LB4 ) can secrete a protease, allowing it to degrade surrounding microbial proteins to obtain organic nitrogen, promoting the germination of *Phragmites australias* seeds, increasing seedling root branches, and improving host plant competitiveness with other plants [79].

In addition, the lipopeptide antibiotic genes in seed endophytes can help hosts resist pathogens. For example, the maize seed endophytic bacterium *Bacillus velezensis* contains biocontrol-related genes (including bioA, bmyB, ituC, fenD, srfAA, srfAB, yngG, and yndJ) that can encode lipopeptide antibiotics, so that they show antifungal activity against pathogens such as *Talaromyces funchoosus*, *Penicillium oxalicum*, and *Fusarium verticillioides* [80]. Endophytic bacteria (*Bacillus subtilis* (KAS2), *Bacillus tequilensis* (KAS3), *Pantoea stewartii* (KAS4), *Pseudomonas aeruginosa* (KAS6), and *Bacillus velezensis* (KAS7)) isolated from pearl millet (*Pennisetum glaucum* L.) showed significant antifungal activity against six kinds of fungal pathogens, among which KAS2, KAS3, and KAS7 contain at least one antifungal lipopeptide gene, and the lipopeptide group of KAS2 is verified to be able to protect seedlings from infection by *Fusarium* sp. [81]. A similar result was also observed for *Lysinibacillus* sp. and *Paenibacillus dendritiformis* colonization within maize seeds [82]. Although most *Bacillus* endophytes in seeds contain lipopeptide genes [83], not all of them are members of the genus, *e.g.*, *Pantoea* and *Pseudomonas* (Table 1).

Fungal diseases can lead to significant decreases in crop yields, *e.g.*, root rot, leaf spot, leaf blight, and pepper wilt caused by *Fusarium oxysporum* [84]. Seed endophytes can effectively resist to these diseases. Verma *et al*. [85] showed that seed endophytes SLB4- *Pseudomonas fluorescens*, SLB6-*Pseudomonas* sp., and SY1-*Pseudomonas* sp. from Phragmites australis can effectively inhibit the infection of rice (*Oryza sativa*) seedlings by *Fusarium oxysporum*; among them, SY1 has the best inhibitory effect and contributes to the growth of roots and stems of rice. This indirect mechanism of promoting rice growth is because seed endophytes can produce HCN (hydrogen cyanide) volatiles and the antifungal genes prnD (pyrrolnitrin) and hcnBC (hydrogen cyanide).

The mechanism by which seed endophytes resist pathogens and promote plant growth involves other pathways, including ROS systems and the production of volatile compounds (Fig. 1). In a study on the use of local seed endophytic microbes to resist fungal diseases of *Urochloa ramosa* L. seedlings, it was confirmed that some seed endophytes can act as signal targets of the redox system (ROS) to regulate genes for inducing host plant defense [86]. In addition, 43% of *Bacillus* seed endophytes in gourds had antagonistic effects against many soil-borne pathogens (*Rhizoctonia solani*, *Fusarium graminearum*) and oomycetes (*Phytophthora capsici*, *Pythium aphanideratum*), mainly through the release of volatile compounds such as acetyl and/or diacetyl that induce plant defense and the production of extracellular ribonuclease [87].

**Resistance to herbivores and insects.** Resistance to herbivores by seed endophytes has been confirmed in many studies, especially for tall fescue seed endophytes that produce mycotoxins (Fig. 1); nonetheless, most of the evidence comes from seed endophytic fungi belonging to a small clade of Clavicipitaceae (Table 1). For example, ergot alkaloids produced by seed endophytes can poison animals (such as cattle) after eating tall fescue [88]. The feeding study found that the mice were more likely to select seeds without infection by the endophytic microbe AR601, and possibly the endophytes were able to release some sort of secondary metabolite, such as ergovaline, which is effective in preventing the feeding of birds and rodents [89]. When red fescue (*Festuca rubra*) contains the seed endophyte *Epichloë festucae*, it is more likely to be infected by the pathogenic fungus *Claviceps purpurea*; interestingly, aphids (*Sitobion* sp.) are not found in most *C. purpurea*-infected plants because *C. purpurea* can produce alkaloids (*e.g.*, ergocristine, ergovaline, ergotamine, and α-ergocryptine). Meanwhile, the endophyte *E. festucae* can also produce high concentrations of ergovaline. These interactions may account for controlling a certain number of insects and are beneficial to the plant growth of red fescue [17]. Furthermore, the endophytic fungus of Epichloë grass, *Epichloë sylvatica*, is transmitted by seeds during asexual reproduction, and it was found that when insect larvae were fed infected *E. sylvatica* of *Brachypodium sylvaticum* leaves, their diet was affected compared to feeding uninfected (E-) insects, suggesting that the endophytic fungus *E. sylvatica* produces a certain chemosensitive substance that increases the resistance of *B. sylvaticum* to herbivorous insects [90].

### Improvement of Plant Resistance to Abiotic Stress

Plants are inevitably exposed to abiotic stresses during growth, and microorganisms present inside the seeds may be critical for improving plant stress resistance (Fig. 1).

**Resistance to heavy metal stress.** Currently, heavy metals cannot be completely degraded in plants, but some seed endophytes can modify the potential toxicity of metals to plants by altering the chemical form of metals through endophyte-mediated oxidation or reduction [91]. As the earliest endophytic microbes colonized by plants, seed endophytes have a potential function in improving plant tolerance to heavy metals and actively induce possible detoxification mechanisms in plants, such as antioxidant systems [92, 93]. There is evidence that the endophytic fungus FZT214 (*Epicoccum nigrum*) of *Dysphania ambrosioides* seeds can increase the chlorophyll and glutathione (GSH) content of host plants at different developmental stages and regulate the tolerance of host plants to Cd stress. On the other hand, the hormone produced by *E. nigrum* colonization in *D. ambrosioides* grown in metal-contaminated environments will also be an important mechanism to promote the growth of host plants [94]. Similarly, the endophyte of *Neotyphodium* in the seeds of *Elymus dahuricus* increased their resistance to the heavy metal Cd by affecting the activities of antioxidant enzymes (including ascorbate peroxidase, catalase, and superoxide dismutase), proline, and malondialdehyde content [95]. In addition, the seed endophytic bacterium *Serratia Nematodima* LRE07 of *Solanum nigrum* L. could enhance plant uptake of essential mineral nutrients by reducing the toxicity of Cd and increasing the activity of antioxidant enzymes [96]. However, the enhancement of this antioxidant system and the mechanisms involved are still not fully understood.

*Lavandula pedunculata* seeds collected in soils contaminated with high concentrations of metals show greater growth ability than those collected in uncontaminated areas, because endophytes colonizing in the seeds may maintain high GSH content under high metal stress to reduce oxidative stress and have potential environmental resilience [97]. When *Agrostis capillaris* grows in Cd/Ni-contaminated soil for a long time, its seed endophytic bacteria, *Bacillus* sp. and *Pantoea* sp., not only have the ability to increase phosphorus release and produce iron carriers but also benefit plant extraction (plants remove heavy metals or toxic compounds from the soil) and plant stability (plants fix metals or reduce the bioavailability of pollutants at different nutrient levels) [56]. In polymetallic contaminated soil, the seed endophyte *Cryptococcus* sp. CBSB78 can increase the absorption of Cd, Zn, and Pb by *Brassica chinensis*, thus reducing the content of heavy metals in soil [98]. Therefore, seed endophytes play an important auxiliary role in the phytoremediation of heavy metals.

**Resistance to salt stress.** Endophytes can effectively improve metabolic disorders of host plants caused by high salt stress and promote plant growth. A recent study showed that *Pantoea* sp. and *Bacillus* sp. members isolated from the seeds of three grasses and four leguminous forages could promote the germination rate of *Medicago sativa* under salt stress [99]. Members of *Bacillus* and *Pantoea* can also produce IAA (indole-3-acetic acid), antagonize fungi, and even have osmotic tolerance [74]. In addition, the endophytic fungus *Fusarium verticillioides* also reduced the effects of salt stress on plants by affecting lipid peroxidation and protein content [100].

**Drought resistance.** Under global climate change, drought is the main obstacle to increasing crop yield. There has been evidence that seed endophytes can improve the drought and heat tolerance of wheat [101, 102]. The endophytic microbe *Kosakonia cowanii* in the seeds of the xerophytic invasive plant *Lactuca serriola* can produce extracellular polysaccharides to maintain soil moisture and thus improve the adaptability of the host plant to drought; in addition, inoculating *Arabidopsis thaliana* with this bacterium can also effectively promote plant growth under drought conditions [103], suggesting that the drought tolerance of seed endophytes is not host species specific. Moreover, there is evidence that xerophytes seem to harbor endophytes resistant to drought, therefore, some researchers focus on isolating strains from drought-tolerant plants and inoculating them into other plant seeds, especially tomato, to improve the plant ability to tolerate drought [104].

Interestingly, climate change may affect seed quality and breeding practices in the agriculture, which in turn may impact seed microbial communities. Because plants are inconsistent in their ability to tolerate drought and heat, continued warming may prolong flowering and alter the depth of dormancy during seed maturation, preventing fundamental assurance of seed quality [105]. For example, *Arabidopsis thaliana* and *Lolium perenne* reduced dormancy times at high temperatures [106, 107]. Similarly, to find more suitable farming methods and planting sites, breeding practices for fighting climate change may change the chemical and physical properties of soil and indirectly lead to changes in endophyte communities [108].

Despite the growing evidence that seed endophytes play an important role in host plants for resisting environmental stress [78, 97, 99, 103], we still face many limitations and challenges in carrying out large-scale applications in the field of sustainable agriculture and natural ecosystems. In particular, there are several questions we must answer thus far, including: how can the target strains be obtained effectively and efficiently? Does a single strain function effectively? Is there any potential interaction between the functional strain and other microbes in the host plant? To date, many studies have explored the function and mechanism of the isolation and then inoculation of seed endophytes back on host plants, which depend on an effective culture technique for target endophytes. Common culture media used to isolate microbes may bias toward easy-growing seed endophytes, such as members of the family Bacillaceae, followed by Paenibacillaceae (Table 1). That is why endophytes in seeds have been continuously discovered by means of high-throughput sequencing techniques, but some species fail to be isolated and cultured from seeds [1, 20, 21]. To obtain a target strain, it may be effective to use selective media to cultivate species (or strains) that adapt to environmental stress [20, 57, 97]. Moreover, the current research focuses on seed bacteria rather than seed fungi. This is likely due to the ease with which seed-associated bacteria can participate in both endogenous phytohormone regulation and external regulation to promote plant growth by enhancing plant adaptation and inhibiting pathogen infestation [69, 75]. In addition, although the use of synthetic communities [109, 110] rather than a single strain should be considered to explore the possible phenotypic characteristics and functions of seed endophytes based on omics, it is a major challenge to understand the interaction between seed endophytes and host plants and other microbes, which is a complex process that may depend on endophyte species, environmental conditions, and gene types of host plants. For example, the interaction of seed fungi and bacteria with arbuscular mycorrhizal fungi (*Rhizophagus irrigularis*) has a negative feedback effect on the growth of *Trifolium repens*, and this negative effect may be competition over resource allocation and temporal priority as well as the release of secondary metabolites [111]. Another study also elaborated that competition for photosynthetic products and plant resources could enable seed-borne *Epichloë* sp. to mitigate the negative effects of *Ustilago bullata* on *Bromus auleticus* [112]. Currently, studies on how seed endophytes interact with other microbes in the host plant are still rare, and more in-depth studies on the mechanisms of their interactions are needed in the future to achieve efficient and sustainable agricultural production by regulating the seed microbiota. Regarding this point, it is helpful to use omics, including transcriptomics, proteomics, metabolomics, genomics, and fluorescent tagging of target strains, to better understand the impacts of seed microbial interactions with other microbes in the host plant.

## Conclusion

As a reproductive organ of plants, seeds are one of the most significant stages in the life history of plants [5] and carry a variety of endophytic microbes that can be transmitted to offspring through parental plants [6]. Undoubtedly, seed endophytes can greatly contribute to host plant resistance to environmental stress. In this article, we first provided a framework for the assembly and function of seed endophytes and discussed the sources and assembly process of seed endophytes; then, we reviewed the impact of environmental factors on the assembly of seed endophytes, and we explored recent advances in the growth promotion and stress resistance enhancement of plants, functioning by seed endophytes under various environmental stressors, including biotic (*e.g.*, pathogens, herbivores and insects) and abiotic factors (*e.g.*, drought, heavy metals and salt). Finally, we also give the limitations and challenges for applications of endophytic seed microbes in the field of sustainable agriculture and ecology. Our review is valuable for understanding the mechanisms of seed endophyte-host plant interactions and has important suggestions for seed endophyte applications, both in the field of sustainable agriculture and in natural ecosystems.

## Figures and Tables

**Table 1:** The function and mechanism for seed endophytes to improve host plant growth and resistance to stress.

Seed endophytes^a^	Host plant	Family^b^	Mechanisms	References
*Bacillus amyloliquefaciens* (B) (Bac)	Rice	Poaceae (C)	Production of gibberellin and ACC deaminase	[69]
*Burkholderia gladioli* (B)(Bur)	Maize	Poaceae (C)	Promotion plant absorption of nutrients	[75]
*Cladosporium cladosporioides* (F) (Cla)	*Suaeda Salsa*	Chenopodiaceae (C)	Secreting certain signal molecules as signal transduction of plant hormones	[70]
*Bacillus* (Bac) *Paenibacillus* (B) (Pae)	Cucurbitaceae family	Cucurbitaceae (C)	Producing auxin IAA	[71]
*Bacillus subtilis* (HYT-12-1) (B) (Bac)	Tomato	Solanaceae (C)	Producing ACC deaminase and organic acid, nitrogen fixation	[72]
*Methylobacterium* (B) (Met)	*Crotalaria pumila*	Fabaceae (C)	There are genes associated with nitrogen and carbon fixation, photosynthesis and oxidative phosphorylation	[77]
*Bacillus* spp. (Bac) *Klebsiella* spp. (Ent) *Staphylococcus* spp. (Sta) *Pseudomonas* spp. (B) (Pse)	*Pachycereus pringlei*	Cactaceae (WP)	Releasing nutrients from rocks; produce organic acids; nitrogen fixation	[73]
*Kosakonia* sp. (Ent) *Pantoea* spp. (Erw) *Xanthomonas* sp. (B) (Xan)	Rice	Poaceae (C)	Producing an iron carrier; dissolve phosphorus	[74]
*Sphingomonas melonis* (B) (Sph)	Rice	Poaceae (C)	Secreting extracellular signal molecules; resistance to pathogens	[78]
*Pseudomonas* spp. (B) (Pse)	*Phragmites australis*	Poaceae (WP)	Production of antifungal metabolites and proteases; resistance to pathogens	[79]
*Bacillus velezensis* (B) (Bac)	Maize	Poaceae (C)	Existence of lipopeptide antibiotic gene and antifungal activity	[80]
*Bacillus subtilis* (B) (Bac)	*Pennisetum glaucum* L.	Gramineae (C)	Existence of antifungal lipopeptide gene	[81]
*Lysinibacillus* sp. (Bac) *Paenibacillus dendritiformis* (B) (Pae)	Maize	Poaceae (C)	Resistance pathogens and improvement seedling growth and development; existence of antifungal lipopeptide gene; synthetic auxin; dissolved phosphate	[82]
SLB4-*Pseudomonas fluorescens* (Pse) SLB6-*Pseudomonas* sp. (Pse) SY1-*Pseudomonas* sp. (B) (Pse)	Rice	Poaceae (C)	Resistance pathogens; promotion root and stem growth; Production of HCN (hydrogen cyanide) volatiles and presence of antifungal genes	[85]
*Methylobacterium* sp. (M3) (Met) *Bacillus amyloliquefaciens* (M4) (B) (Bac)	*Urochloa ramosa* L.	Poaceae (C)	Resistance pathogens and promotion the growth and development of seedlings; existence of antifungal lipopeptide gene	[86]
*Bacillus*(B) (Bac)	Cucurbit	Cucurbitaceae (C)	Releasing acetyl and/or diacetyl; production extracellular ribonuclease; resistance to pathogens	[87]
*Epichloë festucae* (F) (Cla)	*Festuca rubra*	Poaceae (C)	Resistance to insects; producing alkaloids	[17]
*Epichloë sylvatica* (F) (Cla)	*Brachypodium sylvaticu*	Poaceae (WP)	Resistance to insects; producing allelopathic substances	[90]
*Epicoccum nigrum* FZT214 (F) (Did)	*Dysphania ambrosioides*	Chenopodiaceae (WP)	Resistance to heavy metal Cd stress; enhancement of chlorophyll and glutathione content in host plants	[94]
*Neotyphodium* (F) (Cla)	*Elymus dahuricus*	Poaceae (C)	Resistance to heavy metal Cd stress; influence of antioxidant enzymes activity, proline and malondialdehyde content	[95]
*Serratia nematodima* LRE07 (B)(Yer)	*Solanum nigrum* L.	Solanaceae (WP)	Resistance to heavy metal Cd stress; improvement the absorption of nutrients by plants; increasing antioxidant enzyme activity	[96]
*Bacillus* sp. (Bac) *Pantoea* sp.(B) (Erw)	*Agrostis capillaris*	Poaceae (C)	Increasing phosphorus; producing IAA	[56]
*Pantoea* sp. (Erw) *Bacillus* sp. (B) (Bac)	*Medicago sativa*	Fabaceae (C)	Resistance to salt stress; producing IAA, antagonize fungi, and have osmotic tolerance	[99]
*Fusarium*. Verticillioides (F) (Nec)	Soybean	Fabaceae (C)	Resistance to salt stress; influencing lipid peroxidation and protein content	[100]
*Kosakonia cowanii* (F) (Ent)	*Lactuca serriola*	Asteraceae (WP)	Producing extracellular polysaccharides and maintaining soil moisture	[103]

^a^Bac: Bacillaceae; Bur: Burkholderiaceae; Cla: Cladosporiaceae; Pae: Paenibacillaceae; Met: Methylobacteriaceae; Ent: *Enterobacter*iaceae; Sta: Staphylococcaceae; Pse: Pseudomonadaceae; Erw: Erwiniaceae; Xan: Xanthomonadaceae; Sph: Sphingomonadaceae; Cla: Clavicipitaceae; Did: Didymellaceae; Yer: Yersiniaceae; Nec: Nectriaceae. B: Bacteria; F: Fungi.

^b^C: crop; WP: wild plant.
